# Development of an item bank for the EORTC Role Functioning Computer Adaptive Test (EORTC RF-CAT)

**DOI:** 10.1186/s12955-016-0475-x

**Published:** 2016-05-06

**Authors:** Eva-Maria Gamper, Morten Aa Petersen, Neil Aaronson, Anna Costantini, Johannes M. Giesinger, Bernhard Holzner, Georg Kemmler, Anne Oberguggenberger, Susanne Singer, Teresa Young, Mogens Groenvold, on behalf of the EORTC Quality of Life Group

**Affiliations:** Department for Psychiatry and Psychotherapy and Department for Nuclear Medicine, Medical University of Innsbruck, Anichstraße 35, 6020 Innsbruck, Austria; The Research Unit, Department of Palliative Medicine, Bispebjerg Hospital, Bispebjerg Bakke 23, 2400 Copenhagen, Denmark; Netherlands Cancer Institute, Psychosocial Research and Epidemiology, Plesmanlaan 121, 1066 CX Amsterdam, Netherlands; Psychoncology Unit, Sant’Andrea Hospital Sapienza University of Rome, Via di Grottarossa 1035, 00189 Rome, Italy; Department for Psychiatry and Psychotherapy, Medical University of Innsbruck, Anichstraße 35, 6020 Innsbruck, Austria; Department of Medical Biostatistics, Epidemiology, and Informatics, University of Mainz, Saarstraße 21, 55122 Mainz, Germany; Mount Vernon Cancer Centre, Lynda Jackson Macmillan Centre, Northwood, Middlesex, HA6 2RN UK; Department of Public Health, Institute of Health Services Research, University of Copenhagen, Norregade 10, 1165 Copenhagen, Denmark; Department for Psychiatry, Psychotherapy and Psychosomatic Medicine, Medical University of Innsbruck, Anichstraßé 35, 6020 Innsbruck, Austria

**Keywords:** Cancer, Oncology, Health related quality of life, Role functioning, QLQ-C30, Computer-adaptive test

## Abstract

**Background:**

Role functioning (RF) as a core construct of health-related quality of life (HRQOL) comprises aspects of occupational and social roles relevant for patients in all treatment phases as well as for survivors. The objective of the current study was to improve its assessment by developing a computer-adaptive test (CAT) for RF. This was part of a larger project whose objective is to develop a CAT version of the EORTC QLQ-C30 which is one of the most widely used HRQOL instruments in oncology.

**Methods:**

In accordance with EORTC guidelines, the development of the RF-CAT comprised four phases. Phase I involved the conceptualization of RF. In Phase II, a provisional list of items was defined and revised by experts in the field. In phase III, feedback was obtained from cancer patients in various countries. Phase IV comprised field testing in an international sample, calibration of the item bank, and evaluation of the psychometric performance of the RF-CAT.

**Results:**

Phases I-III yielded a list of 12 items eligible for phase IV field-testing. The field-testing sample included 1,023 patients from Austria, Denmark, Italy, and the UK. Psychometric evaluation and item response theory analyses yielded 10 items with good psychometric properties. The resulting item bank exhibits excellent reliability (mean reliability = 0.85, median = 0.95). Using the RF-CAT may allow sample size savings from 11 % up to 50 % compared to using the QLQ-C30 RF scale.

**Conclusions:**

The RF-CAT item bank improves the precision and efficiency with which RF can be assessed, promoting its integration into oncology research and clinical practice.

## Background

In health-related quality of life (HRQOL) research, there is increasing interest in generating computer-adaptive test (CAT) versions of HRQOL measures [1]. CAT facilitates greater measurement precision and may reduce test length by tailoring the set of questions asked to the (estimated) level of functioning or symptom burden of each patient. This makes CAT attractive for research as the increased measurement precision usually entails reductions in required sample sizes making HRQOL data collection more feasible. In addition, the use for HRQOL assessment in clinical practice for detection and tracking of symptoms and as a communication aid has been proven [2]. Hence CAT is especially beneficial for use in clinical routine as it comes with a reduced measurement error in the individual assessment. This characteristic of CAT increases the practicability of HRQOL assessment for clinical use in general, such as for informing physicians and guiding interventions. CAT has already proved efficient for a range of HRQOL constructs such as physical functioning [3] and fatigue [4].

The present paper describes the development of a CAT for role functioning (RF) which is a construct incorporated by all cancer-specific HRQOL instruments and comprises the ability of the individual to fulfil responsibilities typical for a specific age and social setting. Its assessment is quite complex due to the plurality of roles different persons define as relevant and due to the natural fluctuation of such roles over time. However, in the context of health outcome research, it has been argued that the focus needs to be on those aspects of role functioning which are influenced by health conditions and treatment [5]. These include being productive in work and capable of caring for oneself and having a role in immediate and extended social networks [6]. The operationalization of this definition differs across instruments but usually they separate occupational (e.g. work) from social aspects of functioning and assess them on distinct domains. This approach, for example, is applied by the two most widely used HRQOL questionnaires in oncology, the FACT-G (Functional Assessment of Cancer Therapy – General) [7] and the EORTC QLQ-C30 (European Organisation for Research and Treatment of Cancer Quality of Life Questionnaire-Core 30) [8] as well as by the widely used generic (i.e. non disease-specific) SF-36 (Short-Form 36) [9].

Despite agreement on the overall concept the issues that typically are covered in RF measures are quite diverse, ranging from questions on work efficiency to questions on illness acceptance. This is also what poses a special challenge to the development of a CAT for RF as IRT-based measures mostly are based on unidimensional item banks. Although multidimensional CATs can be done, they are highly complex and hence unidimensional structures are usually preferred.

Concurrently, very restricted definitions of RF, for example focusing on physical limitations only or very specific questions such as on reduction in working hours are considered a weakness of existing RF measures [5].

So far, three item banks measuring RF have been successfully developed. The National Institutes of Health-funded PROMIS (Patient Reported Outcomes Measurement Information System) initiative has developed two item banks for RF, one measuring the ability to participate in social roles and one measuring the satisfaction with social roles [10]. Anatchkova et al. [11] developed an item bank for an RF-CAT which comprises occupational, social and family issues and which showed a sufficiently unidimensional structure. All of these item banks are generic and designed to be applicable in a broad range of different health conditions.

The present paper describes the development of a cancer-specific CAT for RF and is part of a larger EORTC Quality of Life Group’s (QLG) project whose goal is to generate a CAT version of the QLQ-C30.

The original QLQ-C30 RF Scale consists of two items (“Were you limited in doing either your work or other daily activities?” and “Were you limited in pursuing your hobbies or other leisure time activities?”). These items concern the domains “work or other daily activities” and “hobbies or other leisure time activities”, respectively, which are also described in the WHO International Classification of Functioning, Disability and Health (ICF) [12]. Thus, these are the domains the new item bank should cover.

The main aims of the study were to:

- Develop a cross-culturally relevant and appropriate item list for the assessment of RF.
- Develop of an item response theory (IRT) calibrated item bank for RF.
- Evaluate the performance of the item bank in CAT simulations using real and simulated data.

## Methods

EORTC item bank development is based on the EORTC guidelines for module development [13] and comprises four phases, namely defining the conceptual framework and conducting a literature search (phase I), operationalization (phase II), pre-testing (phase III) and field-testing and item bank calibration (phase IV). As with EORTC QLG modules these phases include pre-defined development steps and employ a multilingual and cross-cultural approach. The four development phases are summarised below. For further details on the general approach in phase I-III please refer to Petersen et al. 2010 [14], and for phase IV to Petersen et al. 2011 and 2012 [15, 16].

## Conceptual framework and subdomains

In order to ensure that the new RF-item bank is comparable with data collected with the existing (static) version of the QLQ-C30, it needs to cover the same aspects as the QLQ-C30 RF Scale. The item bank should extend the measurement continuum, i.e. allow for the assessment of a broader range of severity of impairment, and increase measurement precision. In addition, the items should fit a unidimensional model in order to be included in the final item bank. The WHO ICF differentiates between limitations in activity and restrictions in participation. To reflect the RF construct as defined within the QLQ-C30 we decided to focus on limitations in activity and considered aspects of participation to be assessed by the social functioning domain of the QLQ-C30.

### Phase I - Literature search

Phase I involved a literature search to collate existing items measuring RF. Searches were applied to the following databases: PubMed, EORTC Item Bank (http://groups.eortc.be/qol/item-bank), ProQolid (https://eprovide.mapi-trust.org/), Psyndex and PsyndexPlus. The search was conducted in September, 2008 applying combinations of the following free text and MeSH-terms: neoplasm*, cancer, role, social, daily, function*, well-being, limitation.

### Phase II - Operationalization

The item list compiled in phase I was refined according to pre-defined selection steps. In each selection step two independent reviewers performed the ratings, which were then compared and discussed in case of disagreement. A third reviewer was involved in case of disagreement, ratings were discussed and then a majority decision was made. Reviewers had expertise in HRQOL, CAT and/or clinical oncology. First, items that were redundant, not compatible with the QLQ-C30 item style, or that assessed issues outside of the scope of conceptual framework were eliminated (step 1). Based on the remaining items, new items in the style of the QLQ-C30 (i.e. a question with a one-week recall period, assessing severity of impairment on a 4-point Likert scale from 1-not at all to 4-very much), were developed (step 2). Step 3 comprised another redundancy rating and a rating of item relevance to the RF construct. In step 4, the remaining items were rated for difficulty (i.e., the level of RF being assessed). Subsequently, they were subjected to QLG internal expert reviews (step 5) before being sent out for international expert reviews (step 6) on the items’ relevance for the assessment of RF, redundancy, clarity, and appropriateness.

### Phase III - Pre-testing

To ensure content validity and the appropriateness of the items for the target population the preliminary item list was pre-tested in an international sample of cancer patients. Inclusion criteria were a cancer diagnosis, age ≥18 years, sufficient command of respective national language, no overt cognitive impairments, and informed consent. Translations were done according to published guidelines by the Translation Office of the EORTC Quality of Life Department [17]. Based on patient feedback, the content and wording of the item list was refined and a preliminary item list to be used in field testing was created.

### Phase IV - Field testing and calibration of the item bank

#### Sample and procedure

The preliminary item list was field-tested in an international sample of cancer patients. Inclusion criteria were the same as in phase III. We aimed at a heterogeneous sample of at least 1,000 patients, which is sufficiently large for the purposes of item calibration [18, 19]. Patients were approached in different oncology treatment settings (e.g., in-patient and outpatient; curative and palliative treatment) in order to cover a broad range of socio-demographic and clinical characteristics as well as different levels of RF impairment. In addition to the preliminary item list, patients completed the QLQ-C30 and answered questions on item relevance, clarity, and appropriateness, which were also presented paper-pencil based.

### Evaluation of dimensionality and local dependence

The items were evaluated to determine if they met the requirements of unidimensionality and local independence using exploratory and confirmatory factor analyses. We were also interested in the potential overlap between the constructs RF and physical functioning (PF). As all patients had completed the QLQ-C30 in phase IV data collection we were able to investigate the factor structure of the new RF items and the physical functioning (PF) items of the QLQ-C30.

Eigenvalues, root mean square error of approximation (RMSEA) <0.10, the Tucker-Lewis Index (TLI) >0.90 and the Comparative Fit Index (CFI) >0.90 were used as criteria in the evaluation of factor structure and model fit [20, 21]. Residual correlations >0.20 served as indicators of local dependence (LD) [22].

### Item bank calibration and evaluation of item fit

Items were checked for monotonicity, i.e. whether the cumulative probability of choosing a given response category or a higher category is non-decreasing with increasing IRT scores, i.e. the better RF, the more likely a response reflecting higher RF should be given. This was done by comparing the average item scores with the sum of the rest scores. Then items were calibrated to a generalized partial credit model (GPCM) [23], a model which allows estimating a discrimination (slope) parameter for each item (i.e. the item’s ability to discriminate between people) and a set of threshold parameters (i.e. the locations on the continuum where the item’s response options are most likely to be endorsed). To assess item fit, S-Χ2 fit statistics [24, 25], the difference between expected and observed responses (bias) and infit and outfit mean-squares (MnSq) were used [26]. Bias is indicated by a root mean square error (RMSE) of ≥1, which would correspond to a difference of one response category. Concerning MnSq-values, primarily large infit and outfit, i.e. >1.3, were regarded as problematic as they indicate poor agreement between observed and expected responses [27]. In addition, to make infit and outfit values less dependent on sample size and variation of responses they can be t-transformed to approximately standard normal distribution. Values outside ±2 (1.96) may be regarded as possibly problematic (95 % CI), and e.g. outside ±2.6 (99 %) as problematic, and outside 3.3 (99.9 % CI) as clearly problematic.

### Differential item functioning

The items then were tested for differential item functioning (DIF), i.e. if items perform differently in certain sociodemographic and clinical subgroups. This was done using ordinal logistic regression [28–30]. Group variables were age, gender, country, cancer site, cancer stage, current treatment, living with a partner/alone, level of education, working/retired/other. Subsequently, for items with DIF it was tested if it affects parameter estimates. The method compares the RF scores obtained with the model which does not account for DIF with a model which does. If the RF scores differ substantially, defined as a difference larger than the median standard error for the RF estimates, this would indicate practically problematic DIF, also termed “salient scale-level differential functioning” [15, 28, 31].

### Evaluation of measurement properties

Finally, the item bank’s performance for CAT measurement was assessed using real and simulated data. CAT simulations to evaluate measurement precision were done using Firestar and were based on the collected data (*N* = 1023). We simulated CATs asking an increasing number of items starting with one and ending with 9. We estimated the RF score based on these CATs, and compared these scores with the score based on all 10 items. As starting item we used the QLQ-C30 RF item with the highest average information. The Expected A Priori (EAP) method was applied for latent trait (theta/θ) estimation.

To evaluate possible savings in sample size, relative validity (RV) of the CATS compared to the QLQ-C30 RF scale in detecting expected group differences was calculated [32]. The RV is the ratio of two test statistics for comparing two (known) groups. We used the t-test statistic for each of the CATs as the numerator and the t-test for the QLQ-C30 RF scale as the denominator – hence an RV >1 indicates that the CAT has greater discriminating power than the QLQ-C30 scale. Known group variables (age, sex, stage, work, therapy, education) were tested if significant for either the CAT or the QLQ-C30 measures. If significant they were used for calculating RVs. This was done based on the collected data.

RV was also assessed on the basis of simulated data. We simulated responses to the items on the basis of RF scores sampled from normal distributions with different means. We compared groups of different sizes and different true effect sizes. For each of the possible settings we ran 2000 simulations. For more details on methods please refer to Petersen et al. 2011 [15] and Petersen et al. 2012 [16]. Statistical packages used were SAS, Parscale [33] and Mplus [34].

## Results

### Phase I: Literature search and item collection

The literature search described previously yielded 122 items on RF from 16 questionnaires.

### Phase II: Operationalization - development of items and expert reviews

**Fig. 1:**
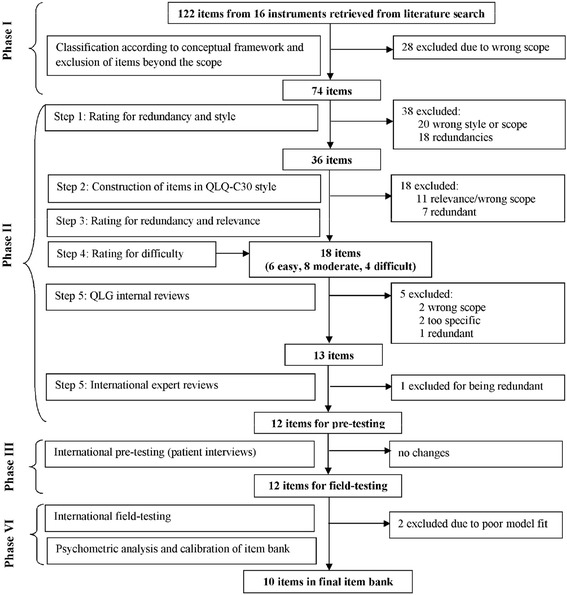
Flowchart of item bank development process

The flowchart in Fig. 1 shows details of the item development process, including numbers of items and reasons for exclusions at each step. In summary, across the selection steps one to four, 61 items were excluded due to incompatible style and lack of relevance for the EORTC RF concept (e.g. *“I have difficulty talking to my boss about the cancer”*), or overlap with other constructs (e.g. *“…dealing with concerns about your family’s ability to cope with caring for you”*) and 27 items were excluded due to redundancy (e.g. *“Were you limited in the kind of work or other activities?”* was considered redundant with the QLQ-C30 RF item on work and other daily activities).The resulting 18 candidate items were then rated for difficulty by three independent experts: six were classified as mainly relevant for patients with good RF, eight as relevant for moderate RF, and four as relevant for patients with poor RF.

Reviewers disagreed on 11–25 % of the items across the developmental steps 1–4. Consensus choices were guided by the policy that patients at different ages and with different diagnoses and stages need to be able to relate to the content of the items.

Upon completion of step 4 the items were reviewed by members of the QLG as well as external international experts. Potential project collaborators are approached at the bi-annual meetings of the QLG. These meetings are frequented by researchers from all over Europe as well as from non-European countries. In total, 14 reviewers from 6 countries (Austria, Australia, Denmark, the Netherlands, Taiwan, and the UK) were involved (steps 5–6). Hence, we were able to obtain reviews from different English speaking countries as well as from European and non-European countries. Based on reviews within the QLG 5 items were excluded as they were rated as not fitting the QLQ-C30 concept of RF, being too specific, or being redundant. Changes that were prompted by the external reviewers concerned clarity (7 items) and appropriateness (1 item). The wording of 4 items was changed accordingly and one item was deleted due to redundancy. The resulting preliminary item list for cross-cultural patient interviews comprised 12 items.

### Phase III: Pre-testing

Patient interviews were conducted in four countries (Austria, Denmark, Italy, UK). The same sample has been used in the development of the emotional functioning CAT [35]. Forty-one patients were interviewed (mean age 63.5 + −11.7 years; 53.7 % female). The sample included patients with a broad range of tumour types and stages. Sample characteristics are provided in Table 1. At this stage, no changes were prompted by patient feedback.

**Table 1:** Patient characteristics

	Sample phase III: Pre-testing (*N* = 41)		Sample phase IV: Field-testing (*N* = 1023)	
Age in years (Mean ± SD)	63.5 (11.7)		61.6 (12.7)	
	No	%	No	%
Sex: Female	22	53.7 %	540	52.8 %
Country				
Austria	10	24.3 %	204	19.9 %
Denmark	10	24.3 %	205	20.0 %
Italy	10	24.3 %	94	9.2 %
UK	11	27.1 %	520	50.8 %
Site				
Breast	8	19.5 %	130	12.7 %
Gastrointestinal	12	29.3 %	199	19.4 %
Testicular, urinary	2	4.9 %	104	10.2 %
Gynaecological	2	4.9 %	97	9.5 %
Head & neck	4	9.8 %	74	7.2 %
Lung	4	9.8 %	90	8.8 %
Other	7	16.9 %	235	23.0 %
Missing	2	4.9 %	94	9.2 %
Tumor stage				
Stage I + II	13	31.7 %	456	44.6 %
Stage III + IV	23	56.1 %	420	41.1 %
Missing	5	12.2 %	147	14.4 %
Current treatment				
Chemotherapy	24	58.5 %	316	30.9 %
Other treatment	2	4.9 %	117	11.4 %
No treatment	14	34.2 %	486	47.5 %
Missing	1	2.4 %	104	10.2 %
Marital status				
Married/Living with partner	26	63.4 %	759	74.2 %
Live alone	12	29.3 %	244	23.9 %
Missing	3	7.3 %	20	2.0 %
Education				
0–10 years	17	41.5 %	376	36.8 %
11–13 years	7	17.1 %	258	25.2 %
14–16 years	7	17.1 %	218	21.3 %
More than 16 years	9	21.9 %	158	15.4 %
Missing	1	2.4 %	13	1.3 %
Work		26.8 %	229	22.4 %
Fulltime	11	26.9 %	93	9.1 %
Part-time	2	4.9 %	564	55.1 %
Retired	24	58.5 %	125	12.2 %
Other	3	7.3 %	12	1.2 %
Missing	1	2.4 %	229	22.4 %

Thus, the provisional item list consisted of 12 items, 9 items covering “work or other daily activities” and 3 items the domain “hobbies or other leisure time activities” (difficulties: 4 easy, 5 moderate, 3 difficult) (see Table 1).

### Phase IV: Field testing and calibration of item bank

#### Sample characteristics and descriptive analyses

In line with the guidelines [13] patients were recruited in the same countries as in phase III. Responses from 1,023 patients were obtained (mean age 61.6 ± 12.7; 52.8 % female) (details can be seen in Table 1). All 12 RF items were answered by 93.4 % of the sample, and only 3.2 % missed two or more items. The respondents generally showed high levels of RF with a mean score of 1.7 (1 = not at all, 2 = a little, 3 = quite a bit, 4 = very much). There was a clear ceiling effect (23 % of patients answered “not at all” to all items), thus patients with poor RF were underrepresented in the sample. However for every item at least 10 patient responses for each response category were present and, thus, response distributions were deemed adequate for analyses and calibration. Generally the patients had very few problems with answering these items (at most 0.4 % reported problems for each item).

### Evaluation of dimensionality and local dependence

The requirements of unidimensionality and local independence were sufficiently met by all 12 items, so they could all be included in IRT analyses (RMSEA = 0.081, CFI = 0.987, TLI = 0.997, residual correlations <0.15). Furthermore, investigation of the factor structure of RF and PF items supported a 2-factor solution. PROMAX rotations indicated that, although items generally loaded strongly on both factors, PF items clearly loaded higher on one factor while RF items loaded higher on the other. Confirmatory analyses showed that a one factor solution would be possible according to TLI and CFI, but RMSEA was not completely satisfying with a value of 0.118 in the one factor CFA and improving significantly to 0.085 in a two factor CFA.

#### Evaluation of item fit

The 12 items were assessed for their fit to a GPCM and for the precision of predicting responses, as well as for redundancy. Two of the 12 items (items ID 7 and ID 12; item text see Table 2) showed a minor deviation from monotonicity of 0.06 corresponding to 2.0 on a 0–100 scale. Wilcoxon tests comparing the item scores in the two RF score groups where a drop occurred resulted in *p* = 0.28, i.e. drop is not significant. Hence, this does not seem to be a genuine deviation but likely random variation.

**Table 2:** Items of phase III and phase IV including difficulties, subdomains, item parameters, and fit indices

Item	Difficulty rating	RF aspects	Slope	Location	Bias	Item fit p-value	Infit	Outfit
Item 1: Have you been limited in completing your household tasks?	Moderate	Work/activities	2.731	−0.784	0.02	0.651	0.95	0.73
Item 2: Have you been limited in doing light housework (e.g. dusting or making the bed)?	Difficult	Work/activities	3.093	−1.059	0.01	0.836	0.94	0.60
Item 3: Have you been limited in doing physically demanding recreational activities (e.g., swimming or cycling)?	Easy	Hobby/leisure	1.907	−0.259	0.03	0.331	0.95	0.79
Item 4: Have you needed assistance in doing your work or daily activities?	Moderate	Work/activities	2.736	−1.019	0.01	0.857	0.93	0.68
Item 5: Have you been limited in doing light recreational activities (e.g., watching TV, playing cards, or reading)?^a^	Difficult	Hobby/leisure	1.986	−1.346	0.00	0.084	1.03	0.66
Item 6: Have you been limited in doing minor household repairs and maintenance (e.g., changing a light bulb or hanging up a picture)?	Moderate	Work/activities	2.169	−1.062	0.01	0.401	0.96	0.68
Item 7: Have you been limited in taking care of personal or household financial affairs (e.g. paying bills)?^ab^	Difficult	Work/activities	1.659	−1.313	0.01	0.744	0.99	0.93
Item 8: Were you limited in doing either your work or other daily activities?^bc^	Easy	Work/activities	3.578	−0.867	0.01	0.821	0.91	0.62
Item 9: Have you been limited in doing heavy housework (e.g., washing floors or vacuuming)?^b^	Easy	Work/activities	3.746	−0.470	0.02	0.098	0.90	0.59
Item 10: Were you limited in pursuing your hobbies or other leisure time activities?^bc^	Easy	Hobby/leisure	2.144	−0.666	0.02	0.211	0.96	0.78
Item 11: Have you been limited in doing your work around the house?	Moderate	Work/activities	Excluded in phase IV					
Item 12: Have you been limited in doing the necessary shopping (e.g., for groceries or clothes)?	Moderate	Work/activities	Excluded in phase IV					

^a^four response categories collapsed to two

^b^significant DIF (item 7: age – higher RF >60 years,item 8: work – higher RF when retired; partner – higher RF if no partner,item 9: country – higher RF in UK + Austria, item 10: gender – higher RF in women,)

^c^QLQ-C30 item

The GPCM converged for an 11-item model. RMSEs between 0.31 and 0.67 indicated some variation in the precision of predicting the item responses, but all were < 1 (i.e. < 1 response category), so were not highly problematic. Furthermore, raw infit values ranging between 0.76 and 1.05 were acceptable. Likewise in the acceptable range were t-transformed infit values ranging between −1.5 and 0.8, except for item ID 12 (see Table 2), for which infit was −4.4 indicating clear problems with redundancy. Therefore it was deleted from the model. Items 5 and 7 (item text can be seen in Table 2) showed reversed thresholds. Although the GPCM would allow the ordering of thresholds to vary across items, reversed thresholds may give rise to counter-intuitive item characteristic (e.g. for some levels of RF answering ‘a little’ or ‘very much’ would then both be more likely than answering ‘quite a bit’). As most responses were in the category “not at all” and then frequencies were decreasing over the categories with the least in “very much”, the disorder may be an artefact caused by having too few responses in these categories. Therefore, response categories were collapsed to two categories (“not at all” vs. rest of categories). Our strategy for dealing with distorted thresholds is based on the approach suggested by Andrich [36] as opposed to the approach suggested by Adams et al. [37].

Finally, this yielded 10 items with good fit indices (S-X2 *p* > 0.05, infits between 0.90 and 1.03, outfits between 0.59 and 0.93, and bias ≤0.03 (which is about one point on 0–100 scale). Item texts and fit indices are shown in Table 2.

#### DIF analyses

DIF analyses showed that items 7–10 demonstrated significant DIF for different variables (details provided in Table 2). Detailed evaluations of the DIF effect indicated that it had only very minor impact on the estimation of RF-scores (all differences in RF scores were less than 0.1 and did not indicate salient scale-level differential functioning). Detailed evaluations of the DIF effect indicated that it had only very minor impact on the estimation of RF-scores; thus no items were deleted (results not shown).

#### Calibration and evaluation of final item bank

As no item had to be excluded due to DIF the final item bank contained 10 items which were calibrated within a GPCM.

**Fig. 2:**
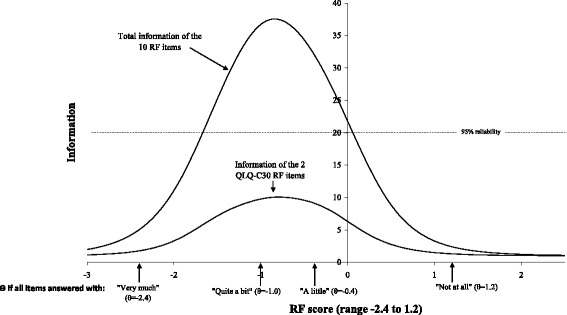
Information curves for the full item bank and for the two QLQ-C30 RF items only, respectively. The RF scores obtained if answering “not at all”, “a little”, “quite a bit”, or “very much”, respectively to all 10 items are indicated at the horizontal axis. Theta scores were estimated using EAP

The measurement precision of the item bank was high. The resulting item bank exhibits excellent reliability (mean reliability = 0.85, median = 0.95, ≥0.90 for 62 %). The score range covered by the item bank is θ = −2.43 (“very much” - lower extreme) to θ = 1.22 (“not at all” - upper extreme) (θ = the characteristic being measured, i.e. a person’s RF score). Particularly for patients answering “a little” or “not at all” to all items, the item pool lacks relevant items at the high end of the role functioning scale continuum. The two QLQ-C30 RF items had total reliability <0.90 (total information ≤10) across the whole continuum (see Fig. 2). The item bank is predominated by work/task related items, however, all available hobby items from the preliminary item list are included. Item parameters as well as different RF aspects covered are shown in Table 2.

**Fig. 3:**
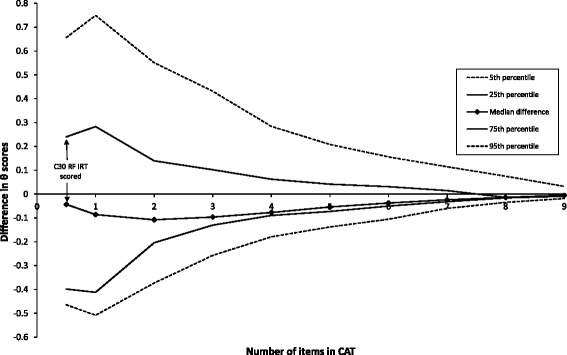
Median and percentiles for differences between theta (θ) estimates based on fixed length CATs and full-length θ

**Fig. 4:**
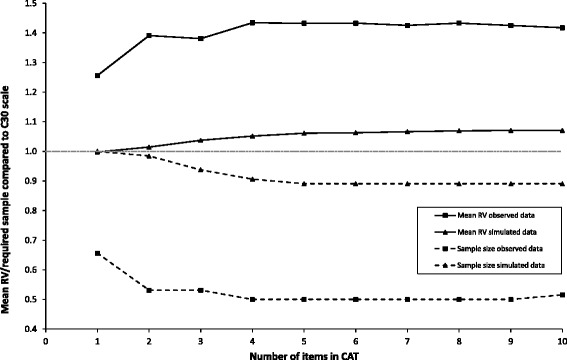
The average relative validity (RV) and relative required sample size using CAT measurement compared to using the QLQ-C30 RF sum scale based on observed and simulated data, respectively

CAT simulations showed that median differences of RF estimates obtained from CAT versions with different lengths and the entire item bank were small (all <0.15 logits) (Fig. 3). As described previously, possible savings in sample size were determined by known group comparisons based on observed and simulated data. Significant known groups were stage (I + II vs III + IV), work (working vs. not working), therapy (currently therapy vs. currently no therapy), and education (below vs. above A-level). Hence, these were used for RV analyses, showing that sample sizes may be reduced without loss of power by asking 2 or more CAT items compared to the static QLQ-C30 RF scale. However, the magnitude of sample size reduction was significantly different when based on observed versus simulated data. The simulated data indicated a maximum of 11 % reduction while the observed data indicated up to a 50 % reduction (see Fig. 4).

## Discussion

Being able to maintain or rebuild ones usual life is an important aspect of coping with a chronic disease such as cancer. Thus, assessing the extent to which a person can fulfil his/her responsibilities is inevitable in the evaluation of oncologic treatments. Furthermore, issues of daily functioning are of utmost importance for cancer survivors, who as a result of rising incidence and decreasing mortality rates are a growing population. For some sites this population even comprises increasingly younger age groups [38, 39].

In HRQOL research in general there is an interest in CAT measures due to their potential of a more effective and precise assessment compared to traditional instruments. Longitudinal studies and monitoring programs in clinical routine may especially benefit from the flexibility of a CAT as they allow repeated assessments with changing sets of questions, supposedly reducing carelessness as well as the impression of redundancy when completing the questions.

In this paper we have reported on the development and initial testing of a CAT for RF within the QLQ-C30 measurement framework. The project resulted in a sufficiently unidimensional item bank comprising 10 items including the two original QLQ-C30 items. We investigated the potential overlap between RF and PF and found that, as expected, they are closely related. However, there is sufficient statistical support for the conceptual separation of the two constructs.

The RF item bank showed high precision for patients with high and moderate RF impairments. A two-item CAT showed higher measurement precision than the original QLQ-C30 RF scale, which also consists of two items. When using CAT with five or more items, there are possible savings of required sample size between 11 and 50 %.

In a previous study [40] based on other data, we had stated that there may be little gain with IRT scoring compared to sum scoring of the QLQ-C30 domains. However, within the EORTC QLG CAT project, we found significant differences in the potential gain from using IRT/CAT across the different domains. Some of the most significant gains were found for the 1 and 2-item scales of the QLQ-C30 (the RF scale being one of those), while for emotional functioning and fatigue CATs with ≤4 items, there was little difference between IRT scoring and sum scoring. Hence, our former conclusion, based on the physical functioning, the emotional functioning, and the fatigue domains only, may have been premature.

A major strength of RF-CAT is the standardized developmental procedure involving international experts from different fields as well as patients themselves enabled the best possible balance between requirements from a psychometric and a practical perspective. This of special importance as the QLQC30 RF scale is designed to assess role impairments in all kinds of cancer patients, i.e. a heterogeneous group concerning sociodemographic and clinical characteristics. Furthermore, as the item bank also comprises the original QLQ-C30 RF items, even data collected prior to this project can be IRT scored now which makes information from earlier and future studies comparable.

As other functioning domains assessed by the QLQ-C30, RF comprises different aspects of the construct, namely work and daily activities and leisure time activities. Within an assessment procedure, the CAT algorithm selects items based on the criterion of maximum information from a statistical perspective. However, to secure the RF construct of the QLQ-C30, content balancing can be included in the algorithm to ensure that the assessment includes both of these aspects of RF, i.e. item selection is based on statistical as well as on content-related reasoning.

A limitation of the item bank is the clear ceiling effect, i.e. it cannot discriminate well between patients with minor RF impairments, which might be of special interest when investigating survivor issues. Construction of well-fitting items relevant for these patients would clearly improve the item bank; however such a task may be difficult. There were no obvious candidate items from the 122 identified from 16 instruments in the original literature search, and therefore, additional work is needed to identify ways to phrase such items.

## Conclusion

Although 10 items form a small item bank, our results suggest that a 2-item CAT based on the item bank may provide an improve measure of RF and enable sample size reductions compared to the static 2-item RF scale of the QLQ-C30. Considering that HRQOL assessments typically cover multiple domains, there is clear benefit from more concise assessment of each single domain. Another advantage of the IRT calibrated item bank is that it will also be possible to create fixed ‘short-forms’ for certain purposes (e.g. screening in clinical routine) without losing comparability with other data collected with other items from the same bank. It should noted that significant differences in estimated required sample sizes were observed, depending on whether known group comparisons were based on simulated or observed data. Hence, the actual gain in terms of sample size reduction may vary across studies, and measurement properties need to be evaluated in independent data. This is currently being done within the ongoing EORTC CAT clinical validation study. The EORTC CAT versions have been integrated into software and will be made available on the EORTC QLG website after completion of the current validation exercise. This will also, hopefully, promote the use of HRQOL assessment in daily clinical practice.

### Ethics, consent and permissions

The study was approved by the ethics committees of the participating centres/countries. All included patients provided informed consent.
